# Lower limb MSK injuries among school-aged rugby and football players: a systematic review

**DOI:** 10.1136/bmjsem-2020-000806

**Published:** 2020-10-28

**Authors:** David Stewart Anderson, John Cathcart, Iseult Wilson, Julie Hides, Felix Leung, Daniel Kerr

**Affiliations:** 1 Life and Health Sciences, Ulster University - Jordanstown Campus, Newtownabbey, UK; 2 Institute of Nursing and Health Research, School of Health Sciences, Ulster University - Jordanstown Campus, Newtownabbey, UK; 3 School of Nursing and Midwifery, Queen’s University Belfast, Belfast, UK; 4 School of Allied Health Sciences, Griffith University, Nathan, Australia

**Keywords:** Epidemiology, Sporting injuries, Adolescent, Football, Australian football

## Abstract

**Objective:**

The objective of this systematic review was to explore the incidence of lower limb musculoskeletal (MSK) injuries sustained by rugby union, rugby league, soccer, Australian Rules and Gaelic football players under 18 years. The review sought to identify the mechanisms and types of injury sustained and to compare between sports.

**Design:**

This systematic review focused on the incidence of lower limb injury in adolescent team sports that involved running and kicking a ball. A literature search of studies published prior to January 2020 was conducted using SportDiscus, Medline and PubMed databases. The Standard Quality Assessment Criteria appraisal tool was used to assess the quality of each article included in the review. Two or more authors independently reviewed all papers.

**Results:**

Sixteen papers met the inclusion criteria; prospective cohort (N=14), retrospective (n=1) and longitudinal (n=1). These studies investigated injuries in rugby union and rugby league (n=10), football (soccer) (n=3), Australian Rules (n=2) and Gaelic football (n=1). There were a total of 55 882 participants, aged 7–19 years old, who reported 6525 injuries. The type, site and mechanisms of injury differed across sports.

**Summary:**

Lower limb injuries were common in adolescent rugby, soccer, Gaelic football and Australian Rules football players, however these studies may not fully reflect the true injury burden where recurrent and overuse injuries have not been considered. There were differences between sports in the mechanisms, types and severity of injury.

## INTRODUCTION

Regular exercise and physical activity have known benefits for children and adolescents,^1–3^ however sports participation can result in injury.^4^ Children and adolescents may be exposed to a range of injuries including skin abrasions, soft-tissue and joint injuries, fractures and head injuries. The most commonly reported injuries are cuts and grazes, falls, hitting or being hit by another person or object and overexertion.^5–7^ The majority of injuries are considered minor to moderate, but some injuries are serious and may have other long-term consequences, affecting both physical and mental well-being.

10.1136/bmjsem-2020-000806.supp1 Supplementary data

Optimal management of sports injuries is influenced by the nature, mechanisms and severity of the injury.^8^ The following definitions of an ‘injury’, a ‘recurrent injury’ and an ‘overuse injury’ have been proposed; an ‘injury’ is a physical complaint experienced by a player during competition that requires medical attention or time loss (TL); a ‘recurrent injury’ is defined as an injury of the same type and location as a previous injury that occurs after a player has returned to competition and training; an ‘overuse injury’ occurs where pain or disability is sustained and where a single injury event cannot be identified.^9 10^

### Injury burden

In terms of quantifying the burden of injury in sports, most researchers have used either a medical attention injury (MAI) or TL (from competition or training) definition, or a combination of both;^11^ for further review, see Bahr.^12^ In simple terms, injury definition can range from an injury that can impact the player’s well-being but does not prevent participation, to an injury that necessitates an absence from competition or training.^13 14^ However, epidemiology studies in sports have moved on. There is now a greater emphasis on overuse injury and illness.^12^ The scope for collecting and reporting data is wider, and should reflect the physical, mental and social well-being of both able-bodied athletes and those with disabilities of all ages, who participate in recreational and elite sports.^15^ For example, Rosen and Heijne^16^ proposed an updated injury incidence model that considered both new and subsequent injuries. This model seeks to expand upon injury surveillance protocols that apply staff-reported, medical attention and TL injuries criteria, to include self-reported data from athletes using text messaging or other web-based apps. The intention is to better understand how subsequent and recurrent injuries may impact athletes, in terms of playing and training with pain and injury.

### Subsequent and recurrent injury

In adults, previous injury has been identified as a significant predictor for the occurrence of subsequent injury. Fulton *et al* ^17^ reported that consecutive anterior cruciate ligament and hamstring injuries were related to previous injuries involving the same tissues. Rodgers *et al* ^18^ stated that Australian Rules footballers, rugby league and rugby union players and soccer players were between 2 and 4.9 times more likely to suffer a subsequent hamstring injury. In adolescent sporting populations, with the exception of soccer, few studies have investigated the association between previous injury and subsequent injury.^19^ Archbold *et al* ^20^ reported that 5% (n=23) of 426 injuries reported in a population (n=815) of 16–18 years old rugby players were considered recurrent; baseline statistics indicate that 552 (n=815) participants reported that they had at least one previous injury from playing rugby union.^20^ With this in mind, it has been suggested that injury surveillance research should provide an evidence-informed context on which to base injury prevention strategies.^21^ Archbold *et al* ^20^ concluded that while the incidence of recurrent injuries in adolescent rugby union players is lower than that reported in professional rugby union players, adolescent players could most likely benefit from the implementation of specific return to play strategies which may help to minimise injury recurrence.

In elite amateur and professional sport, the development of players’ physical attributes (strength, speed, power, endurance and so on) is necessary in order to exploit competitive performance.^22^ Close monitoring of an athlete’s training load is needed in order to reduce the risk of injury from overload,^23^ as part of the return to play phase of rehabilitation, and for a period of time after the athlete has returned to competition.^24^ While it has been established that a dose–response relationship exists between training load, performance and injury in adult athletes, the exact nature of this relationship is not yet fully understood.^25^

In adolescent rugby union however, a similar approach towards training load and performance to that of adult rugby union could have a negative effect on players’ well-being.^26^ Given that adolescent athletes are still growing and developing both physically and mentally, their responses to training are different to that of adults.^27^ As such, strength and conditioning training in adolescents should be adapted to match each stage of growth and development.^28^ In adolescent Australian Rules football, Henderson *et al* ^29^ concluded that appropriate training load management was necessary in order to develop appropriate return to play programmes following injury and that the management of training load in adolescent athletes was necessary to ensure a longer-term participation in sport.^30^

### Overuse injury

The term ‘overuse’ may relate to a mechanism of injury or the cause of an injury. Roos and Marshall^31^ suggested that overuse injuries may be under-reported in injury surveillance studies, given that injury burden is most often measured in terms of TL (one or more days lost) where athletes are unable to compete or train. Athletes with overuse injuries may be managed by medical or training staff and still compete or train, although with some adaptations to training load.^32^ However, in order to implement effective injury management strategies, staff are faced with a number of challenges; correctly identifying where injuries are recurrent (the same injury as before), where an existing injury is exacerbated, or where there are new injuries.^33^

It has been suggested by Bahr^12^ that while consensus statements for studies investigating the prevalence of rugby union and football injuries have been widely accepted,^9 10^ a TL criteria may not appropriately reflect the incidence of athletes who continue to play with pain and reduced functional ability. Clarsen *et al* ^34^ concluded that injury surveillance studies do not fully reflect the potential for overuse injury among athletic cohorts.

The risk of lower limb injury in adolescent sporting populations is high and differs to that of their adult counterparts. Injury is influenced by factors such as the stage of physical and psychological development and the onset of puberty.^35 36^ The highest rates of injury among adolescent male athletes are reported in team sports that include rugby union, rugby league, soccer and football.^36^ The purpose of this systematic review was to identify the common mechanisms and types of lower limb injuries sustained by school-aged populations (12–18 years old at the point of recruitment) across a number of team sports that involve kicking a ball and to compare differences between sports.

## METHODS

Searches of SportDiscus, Medline and PubMed databases were conducted to identify articles published between 2009 and 2020 pertaining to injury prevalence among adolescent participants in team sports that involved running and kicking a ball (rugby union and rugby league, football or soccer, Gaelic football and Australian Rules football).

### Search terms

The following search terms using Boolean operators were applied; incidence OR prevalence OR epidemiology AND musculoskeletal injury AND lower limb AND adolescent OR child OR school-age OR youth OR juvenile OR junior AND sport AND rugby OR football OR soccer OR ‘Gaelic football’ OR ‘Australian Rules football’. Search terms were adjusted for each of the databases.

### Inclusion criteria

Prospective cohort, retrospective, longitudinal and epidemiology studies published in English and studies that met the following inclusion criteria were included:

- Participants were aged 12–18 years old (male or female), where data from males could be extracted. In some instances, participants who were 18 years old at the time they consented to take part had a birthday before the study was concluded; data on these participants were included.
- Sports were included if they involved kicking a ball (rugby union and rugby league, football or soccer, Australian Rules football and Gaelic football).
- Only studies that contained data on frequency, type, site and severity of lower limb injuries were included in the review.

### Exclusion criteria

Non-primary studies such as meta-analyses, systematic and narrative reviews and commentaries and case studies were excluded. Studies that included data for both upper and lower limbs were included, but only if data for lower limb injuries were able to be extracted. Studies with a population aged over 18 years were excluded on the basis that they did not meet the objectives of the review. The full text for each identified study was retrieved and assessed for quality. The reference lists of these identified studies were perused for similar papers that met the criteria.

### Assessment of quality

Studies published from 2009 to 2020 were included in this review in order to reflect the most up-to-date research over a 10-year period. Following an initial search, DK, JC and IW reviewed potential studies retrieved by DSA in order to assess the quality of each article. The full text for each study was retrieved electronically online or via the Ulster University library.

The Standard Quality Assessment Criteria appraisal tool^37^ was used to assess the quality of research papers (online supplemental table 1). This tool evaluates methodological quality and bias risk in both qualitative and quantitative studies. Two authors independently reviewed all papers, while a third arbitrated if inconsistencies were identified.

## RESULTS

**Figure 1:**
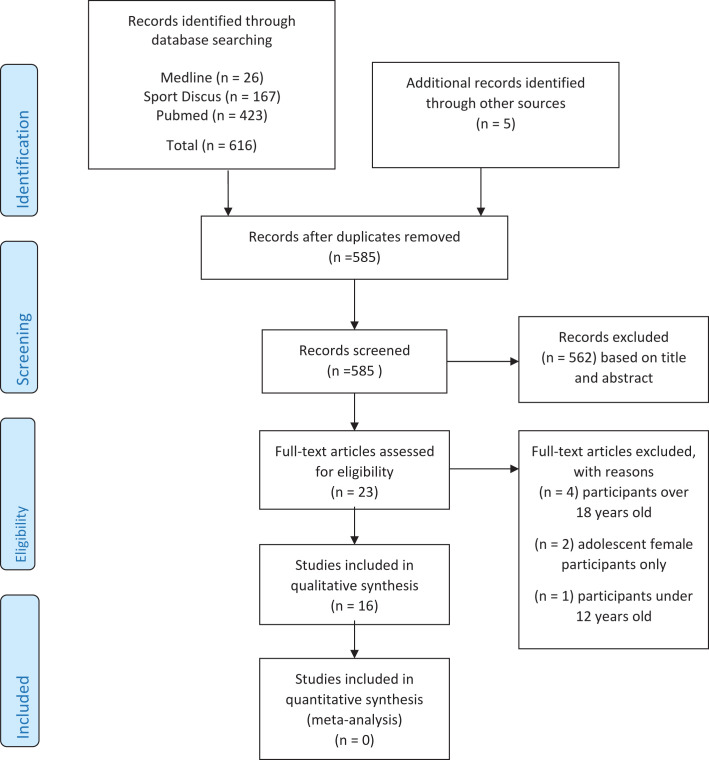
Prisma flow diagram.

A total of 621 articles were retrieved from the database search. Following removal of duplicates, title and abstract screening, and analysis of the text, 16 articles met the inclusion criteria (figure 1), and a data extraction for each study is found in online supplemental table 2. Of the 16 studies, 14 were prospective cohort studies, 1 was a retrospective study and 1 was a longitudinal study. Each study used a paper or online injury recording system with designated individuals (data champions) assigned the responsibility of collection and uploading of data on a weekly basis.

10.1136/bmjsem-2020-000806.supp2 Supplementary data

There were some variations in the duration of data collection in the studies which were included. For example, Barden *et al* ^38^ and Ergun *et al* ^39^ conducted their studies over three seasons. The study by Nicol *et al* ^40^ was carried out over half a playing season. One study (Palmer-Green *et al* ^41^) continued over two seasons. Brown *et al* ^42^ collected data from four 1-week long tournaments during a 2-month period. Kolstrup *et al* ^43^ collected data from three 4-day long tournaments over 3 consecutive years. The other authors collected data over the course of one playing season.^21 44–52^

### Participants

Studies reported data on a total of 55 882 (42 656 males/13 226 females) rugby, soccer, Australian Rules football and Gaelic football players, with an age range of 7–19 years. Several studies included participants from the age of 7 years upwards. In total, 6525 injuries were recorded. Participants were drawn from a number of countries and continents including Africa, Asia, Australia, Europe, Ireland, North America, the Polynesian Islands, Scandinavia, South Africa, South America, Turkey and the United Kingdom (figure 1).

## NATURE AND MECHANISM OF INJURIES

Of the studies involving rugby union and rugby league players, all found that tackling was the most common mechanism of injury. In contrast, all studies involving soccer cited non-contact mechanisms as the most common causes of injury. For the two studies involving Australian Rules football, there were differing results. Lathlean *et al* ^44^ found that contact with other players resulted in most injuries, but Scase *et al* ^48^ concluded that the majority of injuries were of a non-contact nature. One study of Gaelic games was included in this review. O’Connor *et al* ^49^ determined that Gaelic football injuries were mainly of a non-contact nature. In general, it was reported that non-contact injuries occurred because of factors such as biomechanical over-reaching (eg, overstretching causing hamstring strains), overuse and fatigue.

Muscle injuries were found to be more prevalent in soccer,^39 43 46^ Australian Rules football^44 48^ and Gaelic football^49^ than in rugby union. Hip and groin strains and thigh injuries occurred in all sports. Hamstring and quadriceps strains were commonly reported in soccer and Australian Rules football. The most commonly reported injury occurring in adolescent rugby union and rugby league^21 38 40–42 45 47 50–52^ was knee ligament sprain. The rate of injury incidence reported in rugby union and rugby league was significantly higher than that reported in football (soccer), although the incidence rates in Australian Rules football were more comparable with those reported for rugby union. This is likely due to the contact nature of both sports. A high incidence rate of knee ligament injury in rugby union may be because tackling and being tackled by other players is a central component of the game (online supplemental table 3). However, differences in methodological design, injury definitions used and how injury incidence is reported makes it more difficult to compare and contrast findings.

10.1136/bmjsem-2020-000806.supp3 Supplementary data

### Rugby union and rugby league

Nine studies involving rugby union players and one study involving rugby league players were included in this review. Injury rates were reported as the number of injuries per 1000 player hours of exposure (online supplemental table 3). Injury incidence ranged from 10.8/1000 playing hours (ph)^40^ to 77/1000ph.^38^ Nicol *et al* ^40^ proposed that a significantly lower injury incidence rate than that reported in other studies could be due to the fact that data were recorded for matches in one half of a playing season only. While Haseler and colleagues^47^ concluded that injuries sustained in youth rugby union were less frequent and less severe than those sustained in adult rugby union, a study of professional rugby union players by Williams *et al* ^53^ described an injury rate of 88/1000ph; which was comparable to the findings of Barden *et al.* ^38^

### Soccer

There were some differences in how injury incidence rates were reported in the soccer studies included in this systematic review. Ergun *et al* ^39^ and Kolstrup *et al* ^43^ reported injury rates as per 1000 ph, while Read *et al* ^46^ reported injury incidence per club. Overall, a lower injury incidence rate was reported in male soccer players than those reported in the studies involving rugby union players and Australian Rules footballers (online supplemental table 3).

### Australian rules football

Injury incidence rates were reported as the number of injuries per 1000 ph^4 4^ and number of injuries per club.^48^ The injury incidence rate among adolescent Australian Rules football players^48^ was found to be comparable to that of rugby union and rugby league players.^38 41 42 45 47 50–52^

### Gaelic football

One study of adolescent Gaelic games was included in this systematic review. O’Connor *et al* ^49^ reported an injury incidence rate of 9.26/1000ph in Gaelic football players. However, the researchers found that two-thirds of Gaelic footballers continued playing and training after the injury occurred, despite 37.5% of Gaelic football injuries being defined as severe.^49^

## DISCUSSION

Young players of rugby union, rugby league, soccer, Australian Rules and Gaelic football under 12 years are not generally exposed to severe injury. Most injuries to this age group are minor and do not result in a significant TL from competition or training.^47^ As the age of players increased, incidence and severity of injury generally increased. Muscle strains and ligament sprains were primarily classified as minor to moderate injuries. Injuries categorised as severe were fractures, ligament tears or ruptures and meniscus trauma. In some instances, surgery was required.^20 40 49^

All rugby union and rugby league studies included in this systematic review found that injuries in this sport were primarily caused by contact with other players, with the tackle being cited as the most common mechanism of injury.^54^ Both tackler and ball carrier were found to be exposed to injury. In all of the soccer studies, researchers found that non-contact injuries (eg, overuse, fatigue related) were the most common, although injury following collisions with other players and the ground were also reported. With regard to Australian Rules football, the findings of Scase *et al* ^48^ and Lathlean *et al* ^44^ differed somewhat. Scase and colleagues^47^ reported more contact injuries among adolescent Australian Rules football players (49%), compared with 26%.^44^ This may be explained by the fact that Lathlean and colleagues^44^ reported both TL and MAIs, where Scase and colleagues^48^ reported TL injuries only. O’Connor *et al* ^49^ found that lower limb non-contact injuries involving the hip, groin and thigh and the knee were most common (53.4%) in Gaelic football players with lower body injuries accounting for 74.7% of recorded injuries.

A number of intrinsic and extrinsic factors may influence the risk of injury. In explaining why the incidence and severity of injuries in adolescent rugby union and football players generally increased with age, Nicol *et al* ^40^ suggested that this may reflect greater levels of aggression and competitiveness along with morphological adaptation, growth spurts and awkwardness in adolescents, rather than age itself being a risk factor. Rules of the game regarding line-outs and scrums for example vary for different age groups. Injuries were recorded as either TL injuries, MAI or both. Haseler *et al* ^47^ proposed that under-reporting might contribute to a low injury rate in adolescent rugby union players, although differences in methodology can make comparing studies more difficult.

There is an acknowledgement that a greater emphasis on injury prevention, management and rehabilitation strategy is required, given the high incidence of injury and injury recurrence. Barden *et al* ^38^ and Palmer-Green *et al* ^41^ observed that elite level academy rugby union players suffered more injuries than their non-elite and high school counterparts, attributing this to a ‘bigger, faster, stronger’ tendency, or where load was too great (some rugby union players may be playing for school, club and academy). O’Connor *et al* ^49^ suggested that insufficient rehabilitation, which in turn increased injury risk upon return to play, may have contributed to a high (47.3%) injury recurrence rate in adolescent Gaelic footballers.

Similar to adult Gaelic games, more injuries occurred in match settings, in spite of the fact that players spend 6.5 times more time in training sessions, which O’Connor and colleagues^49^ ascribed to the ‘cut and thrust’ of competitive environments.

Participation in rugby union does not appear to place young players at unreasonably greater risk of injury compared with other sports.^55^ Injury rates for child and youth rugby union players have been shown to be lower than that found in the elite and professional game.^54^ Haseler *et al* ^47^ noted that the risk of injury in young rugby union players is extremely low, but the rate and severity of injury increases with age. Of concern is an assertion by Archbold *et al* ^21^ that there is a greater risk of injury in older and heavier schoolboy rugby union players who play with a higher intensity, take greater risks during competitive play and are involved in more matches during the season. While the overall injury rate is lower, adolescent rugby players appear to suffer the same types of injuries as their adult counterparts.

### Confounding factors

With the exception of Archbold *et al* ^21^ and Orr *et al*,^50^ studies did not include history of previous injury prior to participation in their studies. Kolstrup *et al* ^43^ recorded 20 incidences of previous injury among youth soccer players but excluded these incidences from their analysis. Archbold and colleagues^21^ found that of 825 participants, 522 (67%) rugby union players had suffered at least 1 previous sports injury. Orr *et al* ^50^ found that 69% of participants who played rugby league had experienced at least one previous injury (51% from rugby participation). Kolstrup and colleagues^43^ also defined a further 563 injuries as unrelated to soccer and did not include them in their analysis.

Only six studies provided data on re-injury or recurrent injury among their cohorts.^39 41 44 45 48 49^ It is notable that O’Connor and colleagues^49^ concluded that given a high incidence of overuse and recurrent injury in Gaelic football players, closer monitoring of training load and satisfactory completion of appropriate rehabilitation programmes before participants return to play is advisable. Studies examining the impact of previous and recurrent injuries may offer further insight into the phenomenon of the injury burden associated with adolescent rugby union and football populations, and subsequently help inform return to play and injury prevention strategies.

Surveillance studies provide a useful insight into the mechanisms, nature and severity of injuries sustained by adolescent athletes. They may be useful to healthcare professionals, coaches and strength and conditioning trainers involved in developing injury management and prevention strategies.^55^ However, surveillance studies are limited in terms of informing a broader understanding of injury burden among adolescent rugby and football players.^36^ The studies included in this systematic review provided data on injuries that required medical attention or resulted in time lost from competitive play or training sessions, however such studies may not fully reflect how chronic musculoskeletal conditions may impact a player’s physical well-being.

### Injury recurrence and playing with injury

It has been suggested that rugby union and football injuries are often categorised as recurrent and that previous injury is an important predictor for new injury.^56–58^ In contrast, Chambers *et al* ^59^ found in a study involving 704 male rugby union players aged 13–35 and older that previous injury was not a predictor of injury incidence. The authors, however, reported that rugby union players who were playing with an existing injury had a 46% higher risk of suffering a new injury.

Similarities appear to exist between adolescent and elite rugby players in terms of mechanism of injury, particularly in tackle situations.^21 54^ Given that players continue to train and compete while injured, Clarsen *et al* ^60^ proposed the implementation of a qualitative approach to injury surveillance, where the emphasis is on gaining an appreciation of pain, functional deficit and ability to perform in the context of overuse. Observational studies using interviews or questionnaires could be used to collect qualitative data on the experiences of injured players who are playing with pain and injury.

Identifying when an overuse injury begins may not be straightforward. Clarsen *et al* ^60^ found that only 9% of injuries could be classified as ‘new’ with most participants having previous injuries or symptoms at the beginning of the study. In regard to recording surveillance data during the playing season, difficulties may arise when different healthcare professionals enter data differently in relation to re-injury or recurrent injury.^61^ Shrier *et al* ^62^ proposed a model that could be used for the analysis of subsequent injuries in sport (M-FASIS; multistate framework for the analysis of subsequent injury in sport). It was proposed that this model could help researchers to track injuries, determine mechanisms of injury and help healthcare professionals to define appropriate prevention and rehabilitation programmes for their athletes. However, according to the authors, the framework is not without some issues. It may prove difficult to implement these types of injury tracking models in schools and community sports settings where access to suitably experienced or qualified staff or sufficient funding may be an issue. Nevertheless, such models appear to provide a useful means of tracking injury burden among athletic populations and could be beneficial for developing injury prevention strategies in adolescent rugby and football players.^16 63^

## CONCLUSION

Injury incidence studies indicated that lower limb injuries were common in adolescent rugby union and rugby league, soccer, Gaelic Athletic Association (GAA) and Australian Rules football players. Ligament injuries of the knees and ankles, thigh strains and contusions were the most common types of lower limb injury sustained by players of all codes of football. Injury rate and severity of injury increase with age. Most injuries in rugby union were contact injuries, sustained during tackling. Both the tackler and ball carrier were exposed to the risk of injury. In soccer, Australian Rules football and Gaelic football, injuries were predominantly of a non-contact nature. Injury incidence studies provide a useful insight into injury risk in adolescent athletes but may not fully reflect the phenomena of injury burden among injured adolescent rugby, soccer and football players.

While the majority of injuries are minor or moderate, some are very serious and may have long-term consequences for adolescent athletes. Apart from the impact on their school studies, some injuries may prevent adolescents from participating in sport in the future. There may be health consequences later on, affecting both physical and mental well-being. Injuries place a burden on families as well as the healthcare sector. Further research investigating the confounding factors (previous and recurrent injury, playing with pain or injury), and mental well-being of injured adolescent rugby and football players is recommended.

**Summary box**

**What is already known**

- Children and adolescents are exposed to a range of injuries. Most are considered minor to moderate, but some injuries are serious and can have long-term consequences.
- Most epidemiological studies use a medical attention injury or time loss definition (or both) when reporting the incidence of injury.
- Elite level adolescent rugby union, rugby league, soccer, Gaelic football and Australian Rules football players suffer injuries similar to that of their adult counterparts, in terms of nature and severity, though with less frequency.

**What are the new findings—implications for future research**

- Adolescent rugby and football players who suffer serious injury may suffer both physically and mentally. Qualitative studies may be helpful in ascertaining the impact long-term or serious injury has on the mental well-being of adolescent rugby and football players.
- It is recommended that studies investigating the incidence of injury in adolescent rugby and football should consider confounding factors such as previous injury, recurrent injury and playing with pain and injury.
