# Supplementary material for: Lower limb MSK injuries among school-aged rugby and football players: a systematic review
Source: BMJ Open Sport Exerc Med. 2020 Oct 28;6(1):e000806. doi: 10.1136/bmjsem-2020-000806 (PMC7642221; doi:10.1136/bmjsem-2020-000806)
Supplement: Supplementary data [file bmjsem-2020-000806s001.pdf]

Table 3. Injury Incidence and most common lower limb injury and injury type

Study	Incidence	Injury site	Type of injury
Archbold (R)	29.06	knee/ankle/hamstring	sprain/strain
Barden (R)	77 (AASE) 34 (non-ASSE)	ankle/knee/thigh	sprain/strain
Brown (R)	47.9 (all) 23.1 (TL)	not specified	sprain/strain
Haseler (R)	24 (all) 49.3 (U17)	knee/ankle	joint/ligament
Leung (R)	31.8	knee/ankle and foot	ligament/joint
Leung (R)	23.7 (all) 49.2 (14yo)	knee/ankle	ligament/joint
Nicol (R)	10.8	knee/calf muscle	sprain/strain
Orr (R)	37.1	ankle/knee	bruises/contusions hematoma/sprain
Palmer-Green (R)	47 (academy) 35 (schools)	knee/ankle/heel	sprain/contusion lacerations/lesions
Sewry (R)	28.8 (all) 18.9-38.6	not specified	joint/ligament
Ergun(F)	18.4 (MAI) 12.1 (TLI)	thigh/hip & groin	strains/contusions
Kolstrup (F)	13.1 (boys) 20.3 (girls)	ankle/foot/knee/leg	contusion/dislocation sprain/strain
Read (F)	1.32 (p/player) 2.14 (U18)	knee/ankle/quads	strain/sprain
Lathlean (AF)	37.2	thigh/ankle/knee hip & groin	muscle/inflammation and swelling
Scase (AF)	19.3 (p/club)	ankle/hamstring	joint/muscle strains
O'Connor (GF)	9.26	knee/hamstring/ankle	strain/sprain

Injury incidence per 1000 hours. Mean is specified, unless otherwise stated. TLI – time loss injury.

MAI – medical attention injury. R – rugby, F – football, AF – Australian rules football, GF – Gaelic Football.
