# Supplementary material for: Lower limb MSK injuries among school-aged rugby and football players: a systematic review
Source: BMJ Open Sport Exerc Med. 2020 Oct 28;6(1):e000806. doi: 10.1136/bmjsem-2020-000806 (PMC7642221; doi:10.1136/bmjsem-2020-000806)
Supplement: Supplementary data [file bmjsem-2020-000806s002.pdf]

Standard Quality Assessment Criteria - Checklist for assessing the quality of quantitative studies

Key: Yes (2), Partial (1), No (0), N/A (not applicable)

Criteria / study	**Archbold**	**Barden**	**Brown**	**Ergun**	**Haseler**	**Kolstrup**	**Lathlean**	**Leung (JSS)**	**Leung (JSAMS)**	**Nicol**	**O'Connor**	**Orr**	**Palmer-Green**	**Read**	**Scase**	**Sewry**
1. Question / objective sufficiently described?	2	2	2	2	2	2	2	2	2	2	2	2	2	2	2	2
2. Study design evident and appropriate?	2	2	2	2	2	2	2	2	2	2	2	2	2	2	2	2
3. Method of subject / comparison group (if applicable) characteristics sufficiently described?	2	2	2	2	1	1	1	1	2	2	2	2	1	1	2	1
4. Subject (and comparison group, if applicable) characteristics sufficiently described?	2	1	1	2	1	1	1	1	1	1	2	1	1	2	0	0
5. If interventional and random allocation was possible, was it described?	N/A	N/A	N/A	N/A	N/A	N/A	N/A	N/A	N/A	N/A	N/A	N/A	N/A	N/A	N/A	N/A
6. If interventional and blinding of investigators was possible, was it reported?	N/A	N/A	N/A	N/A	N/A	N/A	N/A	N/A	N/A	N/A	N/A	N/A	N/A	N/A	N/A	N/A
7. If interventional and blinding of subjects was possible, was it reported?	N/A	N/A	N/A	N/A	N/A	N/A	N/A	N/A	N/A	N/A	N/A	N/A	N/A	N/A	N/A	N/A
8. Outcome and (if applicable) exposure measures well defined and robust to measurement / misclassification bias? Means of assessment reported?	2	2	2	2	1	1	2	2	2	2	2	2	2	2	2	2
9. Sample size appropriate?	2	2	2	2	2	2	2	2	2	2	2	1	2	1	2	2
10. Analytical methods described / justified and appropriate?	2	2	2	2	2	2	2	2	1	1	2	2	2	1	2	2
11. Some estimate of variance is reported for main results?	2	2	2	2	2	2	1	2	2	0	2	1	2	1	1	1
12. Controlled for confounding?	1	0	0	0	0	1	0	0	1	0	0	0	0	0	0	0
13. Results reported in sufficient detail?	2	2	2	2	2	2	2	2	2	2	2	2	2	1	2	1
14. Conclusions supported by results?	2	2	2	2	2	2	2	2	2	1	2	1	1	1	1	2
**TOTAL SUM / TOTAL POSSIBLE SUM (28) TPS = 28 - (N/A x2)**	21 / 22	19 / 22	19 / 22	20 / 22	17 / 20	18/22	17 / 22	18 / 22	19 / 22	15 / 22	20 / 22	16 / 22	17 / 22	14 / 22	16 / 22	15 / 22
