# Supplementary material for: Lower limb MSK injuries among school-aged rugby and football players: a systematic review
Source: BMJ Open Sport Exerc Med. 2020 Oct 28;6(1):e000806. doi: 10.1136/bmjsem-2020-000806 (PMC7642221; doi:10.1136/bmjsem-2020-000806)
Supplement: Supplementary data [file bmjsem-2020-000806s003.pdf]

**Author and title of paper**	Study design	Methods	Data Collection	Timeframe	Number of participants	Age Range	Total number of injuries	Number of participants who received an injury	Total number of lower limb injuries	Participants who received more than one injury	Defined as a time loss injury	Defined as medical attention injury only	Soft tissue (strain or tear)	Bone (fracture)	Joint (capsule, meniscus and ligament injury)	Hip / Groin	Knee	Ankle / Foot	Thigh	Calf / Lower leg	History of at least one previous sports injury	Most common injury MOI / region	Recurrent injury / re-injury
**Archbold et al. (2017) RISUS study: rugby injury surveillance in Ulster schools. BJSM, 51, 600 - 606. Doi 10.1136/bjsports-2015-095491**	Prospective Injury Surveillance Study	Online reporting system for recording injury data	Data imputed weekly by data champion	One playing season (2014/15)	825	16 - 17	426		171		n =208 (28+ days) 90% (7+ days)		65	38	146	16	56	47	37	14	552	Tackle / Collision lower limb	
**Barden et al. (2018) Epidemiology of injury in elite English schoolboy rugby union: A 3-year study comparing different competitions. Journal of Athletic Training, 53(5), 514 - 520. Doi 10.4085/1062-6050-311-16**	Retrospective Cohort Study	Injury surveillance study using Orchard's Sports Injury Classification System	Medical team recorded all injuries pitchside or in clinic	Three seasons (Aug 12 - May 2015)	132	16 - 19	103	64			103		26	2	33							Tackle / Collision lower limb (by a small margin)	
**Brown et al (2012) The incidence and severity of injuries at the 2011 South African Rugby Union (SARU) youth week tournaments. SAJSM, 24(2), 49 - 54. Doi**	Prospective Cohort Study	Injury surveillance using paper-based questionnaire	Data collected by data champion at each event, submitted to tournament doctor	Four 1-week events during June and July 2011	1804	12 - 18	189	185		4	91	87 (11 not diagnosed)	U16 - 14% U18 - 16% U18 - 0%	U16 - 3% U18 - 6% U18 - 2%	U16 - 31% U18 - 32% U18 - 37%							Muscle / tendon & joint / ligament	
**Ergun et al. (2013) Injuries in elite youth football players: a prospective three year study. AOTT, 47(5), 339 - 346. Doi 10.3944/AOTT.2013.3177**	Prospective longitudinal 3 year study	Injury surveillance over 3 years with one group of players	Data imputed post match by designated data champion	Three seasons (05/06, 06/07, 07/08)	52	16 - 17	44	24	79.50%		29	15	17	0	4	8	2	2	8	0		Non-contact / lower limb	11
**Haseler et al. (2010) The epidemiology of injuries in youth community rugby union. BJSM, 44, 1093 - 1099. Doi 10.1136/bjsm.2010.074021**	Prospective Cohort Study	Injury surveillance using paper-based questionnaire	Data imputed weekly by designated first aider / coach	One season (9 months)	210	7 - 17	39	33		4							4.9 / 1000ph	2.4 / 1000ph				Tackling (tackler and ball carrier) knee.	
**Kolstrup et al. (2016) Injuries during football tournaments in 45,000 children and adolescents. European Journal of Sports Science, 16(8), pp. 1167 - 1175**	Cohort Study	Football related medical attention injury data recorded each day of event	Data collected by data champions at each event	Three Dana Cup (four day) tournaments (2012 - 2014)	45606	11 - 19	2518	1831 (1091 boys) (740 girls)				1831	Spr / Str 11-15yo 2.74 (1000 ph) 16 - 19yo 3.53 (1000ph)	11 - 15yo 1.14 (1000 ph) 16 - 19yo 0.53 (1000 ph)		11 - 15yo 1.62 (1000 ph) 19 - 19yo 1.95 (1000ph)	11 - 15yo 1.97 (1000 ph) 16 - 19yo 2.77 (1000 ph)	11 -15yo 3.29 (1000 ph) 16 - 19yo 4.40 (1000 ph)			20 (excluded from study)	Lower limb (older boys), shoulder & upper limb, younger boys)	563 (excluded from study)
**Lathlean et al. (2018) The incidence, prevalence, severity, mechanism and body region of injury in elite junior Australian football players: A prospective cohort study over one season. JSAMS. Doi 10.1016/j.jsams.2018.03.002**	Prospective Cohort Study	Online reporting system for recording injury data	Injuries reported by players and medics, recorded by physiotherapists	Six months (2014 playing season)	562	16 - 18	1192	449	720	28			52	0	91	109	140	112	193	116		Contact (player / collision) / lower limb	36
**Leung et al. (2017) Injuries in Australian school level rugby union. JoSpSc, 35(21), 2088-2092. Doi 10.1080/02640414.2016.1255771**	Prospective Observational Study	Injury surveillance using paper-based questionnaire	Data inputed weekly by physiotherapist / sport trainers	One eight week playing season	480	17 - 18	80	76		2			8	8	27	2	7	7		2		Tackling (tackler and ball carrier) lower limb	2
**Leung et al. (2017) Epidemiology of injuries in Australian school level rugby union. JSAMS, 20(2017), 740 - 744. Doi 10.1016/j.jsams.2017.03.006**	Prospective Observational Study	Injury surveillance using paper-based questionnaire	Data imputed weekly by designated first aiders	One playing season (2016)	3585	9 - 18	332	310	79	22			13	0	36	9	27	27	8	7		Tackling (tackler and ball carrier) upper limb	
**Nicol et al. (2010) Rugby union injuries in Scottish schools. JPH, 33(2), 256 - 261. Doi 10.1093/pubmed/fdq047**	Prospective Cohort Study	Injury surveillance using paper-based questionnaire	Data collected by designated member of staff	Four months (2009)	470	11 - 16	37		8		8	29	6	5	11	0	5	0	0	3		(tackler and ball carrier)	
**O'Connor et al. (2015) Epidemiology of injury in male adolescent games. JSAMS, 19(2016), 384 - 388. Doi 10.1016/j.jsams.2015.06.002**	Prospective Cohort Study	Injury surveillance based on NCAAISS	Data collected by designated data champion	One year	292	14 - 17	125	95		25			GF - 28% H - 14%	GF - 4% H - 6.1%	GF - 28% H - 16.3%	GF - 13.3% H - 10%	GF - 18.7% H - 20%	GF - 12.7% H - 12%	GF - 28% H - 8%	GF - 6.7 H - 6%		Non contact / lower limb (though	GF - 47.3% H - 33.3%
**Orr et al. (2016) Incidence and characteristics of injuries in elite Australian junior rugby league players. JSAMS 19, 212 - 217. Doi 10.1016/j.jsams.2015.03.007**	Prospective cohort Study	Injury surveillance using paper-based questionnaire	Data collected by designated person, follow-up completed by physio, rehab pro or doctor	One Season (2012)	122	14 - 17	109	63	48	15			8	4	29		10	14	12	12	69% (51% from Rugby)	Tackling (tackler and ball carrier) lower limb	
**Palmer-Green et al. (2013) Match injuries in English youth academy and schools rugby union. An epidemilogical study. AJSM, 41(4), 749 - 755. Doi 10.1177/0363546512473818**	Prospective Observational Cohort Study	Injury Surveillance based on Orchard's coding system	Data imputed weekly by first team coach, nurse or physician	Two seasons (2006/07 and 07/08)	472	16 - 18	243						17	6	38		21					Tackling (tackler and ball carrier) lower limb	28
**Read et al. (2017) An Audit of injuries in six english professional soccer academies. J Sp Sci. 36(13), 1542 - 1548. Doi 10.1080/02640414.2017.1402535**	Prospective Cohort Study	Injury surveillance using questionnaire based injury form	Data recorded by club doctor	one season (2014/15)	608	11 - 18	804						162	23	131	102	161	147	125	34		Non-contact / lower limb	
**Scase et al. (2012) The epidemiology of injury for an elite junior Australian football cohort. JSAMS 15, 207 - 212. Doi 10.1016/j.jsams.2011.12.002**	longitudinal Cohort Study	Injury surveillance questionnaire, using Orchard classification system	Data imputed post match by designated data champion	One season	532	15 - 18	256		164							80	30	54				Non-contact / lower limb	12.8% (2.2 per club)
**Sewry et al. (2019) Seasonal time-loss match injury rates and burden in South African under-16 rugby teams. JSAMS, 22, 54-58. Doi 10.1016/j.jsams.2018.06.007**	Prospective Cohort Study	Time loss injuries recorded using qustionnaire based data collection form	Data collected by designated medical person, minimum 24 hrs time loss outcome	One playing season (2017)	130	15 - 16					33				12.2 / 1000 ph							Tackling (tackler and ball carrier) lower limb	
